# Efficacy and Safety of Hepatectomy Performed with Intermittent Portal Triad Clamping with Low Central Venous Pressure

**DOI:** 10.1155/2013/297971

**Published:** 2013-12-12

**Authors:** Serdar Topaloglu, Kıymet Yesilcicek Calik, Adnan Calik, Coskun Aydın, Sema Kocyigit, Huseyin Yaman, Dilek Kutanis, Erdem Karabulut, Davut Dohman, Asim Orem, Mithat Kerim Arslan

**Affiliations:** ^1^Department of Surgery, School of Medicine, Farabi Hospital, Karadeniz Technical University, 61080 Trabzon, Turkey; ^2^Department of Nursing, School of Health Sciences, Farabi Hospital, Karadeniz Technical University, 61080 Trabzon, Turkey; ^3^Department of Biochemistry, School of Medicine, Farabi Hospital, Karadeniz Technical University, 61080 Trabzon, Turkey; ^4^Department of Anesthesiology, School of Medicine, Farabi Hospital, Karadeniz Technical University, 61080 Trabzon, Turkey; ^5^Department of Biostatistics, School of Medicine, Hacettepe University, Sıhhiye, 06100 Ankara, Turkey

## Abstract

*Background*. This retrospective study was designed to investigate the efficacy and safety of intermittent portal triad clamping (PTC) with low central venous pressure (CVP) in liver resections. *Methods*. Between January 2007 and August 2013, 115 patients underwent liver resection with intermittent PTC. The patients' data were retrospectively analyzed. *Results*. There were 58 males and 57 females with a mean age of 55 years (±13.7). Cirrhosis was found in 23 patients. Resections were performed for malignant disease in 62.6% (*n* = 72) and for benign disease in 37.4% (*n* = 43). Major hepatectomy was performed in 26 patients (22.4%). Mean liver ischemia period was 27.1 min (±13.9). The mortality rate was 1.7% and the morbidity rate was 22.6%. Cumulative clamping time (*t* = 3.61, *P* < 0.001) and operation time (*t* = 2.38, *P* < 0.019) were significantly correlated with AST alterations (D-AST). Cumulative clamping time (*t* = 5.16, *P* < 0.001) was significantly correlated with D-ALT. Operation time (*t* = 5.81, *P* < 0.001) was significantly correlated with D-LDH. *Conclusions*. Intermittent PTC under low CVP was performed with low morbidity and mortality. Intermittent PTC can be safely applied up to 60 minutes in both normal and impaired livers.

## 1. Introduction

Operative blood loss is one of the main factors associated with perioperative prognosis of patients undergoing liver resection [1–6]. Bleeding during liver resection generally occurs in the dissection phase, during the parenchymal transection or during the revascularization phase of the procedure. Especially resection of lesions in close proximity or infiltrating major vascular structures (i.e., the cava hepatic junction) or an extended hepatectomy can be unpredictably complicated by life-threatening hemorrhage. Various strategies to reduce intraoperative bleeding during hepatectomy have been described in the literature [7]. Afferent or complete devascularization before parenchymal transection and precise hemostasis during parenchymal transection with the assistance devices (including ultrasonic dissection, heat coagulation, or bipolar vessel sealing), hepatectomy performed under low central venous pressure (CVP)s and temporary occlusion of blood inflow with or without outflow control are important strategies for prevention from blood loss.

Temporary occlusion of blood inflow of the liver, also called Pringle maneuver (PTC, portal triad clamping), is simply applied without complex anesthetic management [7]. However, total vascular exclusion of the liver including PTC and occlusion of the inferior vena cava below and above the liver may need complex surgical technique and anesthetic management [7, 8]. Both types of vascular occlusion techniques are effective in limiting bleeding, but they also produce liver ischemia. To minimize ischemia-reperfusion injury to the liver remnant, intermittent PTC is commonly used [7]. It is not yet fully known how much vascular occlusion influences the amount of parenchymal bleeding, the rate of hepatocyte damage, subsequent recovery, and the surgical outcome. The efficacy and safety of intermittent PTC during liver resection under low CVP were evaluated in the present study. Outcome variables included the degree of ischemia-reperfusion injury, intraoperative blood loss, alteration of liver functions, and the incidence and severity of postoperative complications.

## 2. Patients and Methods

One hundred and thirty-six patients who were candidates for elective liver resections between January 2007 and August 2013 were considered eligible for this study. Exclusion criteria were an age of 16 years or younger, surgery on the liver without parenchymal resection (including inoperable cases, *n* = 9), and liver resection performed without intermittent PTC (*n* = 12). Overall, 115 patients had undergone 116 liver resections. The patients' data were prospectively recorded and retrospectively analyzed. The study was approved by the institutional committee on human subjects.

### 2.1. Patient Selection

All patients considered for resection underwent preoperative assessment. Laboratory tests included liver function tests, coagulation tests, and measurement of serum creatinine and electrolytes. Each patient was preoperatively evaluated by an abdominal triphasic computed tomographic scan to plan the liver surgery. For malignant diseases, patients were considered operable if all diagnosed tumors could be treated by radical resection with macroscopically negative surgical margins and a sufficient future liver remnant. Functional liver status was evaluated by using the Child-Pugh-Turcotte (CPT) score. Patients with CPT score greater than 7 and model for end-stage liver disease score greater than 16 were considered for the liver transplantation program instead of the liver resection procedure. Sufficient future liver remnant is assessed with the help of manual volumetry from CT images. Additionally, 2 of 3 major hepatic veins were preserved during major liver resection in patients with chronic viral hepatitis or presence of cirrhosis.

### 2.2. Hepatectomy and Perioperative Care

Central vein catheterization was performed routinely and central venous pressure (CVP) was maintained at less than 5 cm H_2_O during liver resection. Conventional liver resection was performed through a J incision. Extraparenchymal control of ipsilateral inflow and outflow was attempted before resection. Resection was performed under intermittent PTC in general (*n* = 115/127). Intermittent PTC in cycles of 15/5 min of clamp/unclamp times was used in all patients. Liver transection was performed with the combination of clamp crushing method and vessel sealing system (Ligasure, Covidien AG, CA, USA) [9]. We used Bismuth's terminology for hepatectomy (segmental and sectorial division of liver parenchyma) in this study [10]. Major hepatectomy was defined as the resection of three or more segments. All patients received antibiotic prophylaxis. Hemorrhage during liver resection was replaced with fresh frozen plasma (*n* = 93/116). Transfusion with erythrocyte suspension was required in 37 of 116 resections. Extubation of the patient in the operating room was achieved in 113 of 115 patients. Patients with uneventful operative course were transferred to the surgical ward after extubation (*n* = 113/115). Prophylactic daily subcutaneous injection of low-molecular-weight heparin sodium was started on postoperative day (POD) 0.

### 2.3. Postoperative Followup and Data Collection

Patients received standardized pulmonary care a day after extubation [9]. Patients were seen daily by the surgical team until hospital discharge. Parameters on postoperative liver function (i.e., INR, levels of bilirubin, liver transaminases (aspartate aminotransferase, AST; alanine aminotransferase, ALT), *γ*-glutamyltransferase (GGT), alkaline phosphatase (ALP), and lactate dehydrogenase (LDH)) were measured preoperatively and daily during the first postoperative week. Duration of warm ischemia and operative time were recorded. Intraoperative blood losses were calculated by adding the blood volume into the suction canister to the blood loss as calculated by weighing the sponges. Patients were observed until the day of discharge. Postoperative complications including pulmonary complications, wound infection, biliary leak, deep venous thrombosis, and liver failure were recorded. All postoperative complications determined in the study group were classified according to *Dindo's* description [11]. Routine abdominal ultrasound was carried out in any patient with a suspected infected collection. All fluid collections were drained percutaneously with bacteriologic cultures. The length of hospital stay was recorded.

### 2.4. Statistical Analysis

Data were expressed as mean (± standard deviations) or median (range). Data were collected to a computer using SPSS software (version 11.0; SPSS Inc., Chicago, IL). To assess the hepatic injury response, the delta (D)-AST (maximum level minus preoperative level) and D-ALT were calculated. To assess the ischemic insult, the D-LDH was calculated. To identify factors affecting D-AST, D-ALT and D-LDH various clinical variables were evaluated by multiple linear regression analysis. Comparative analysis of categorical variables was performed using Mann-Whitney *U* test and Kruskal-Wallis test. Comparative analysis of numeric variables was performed using Spearman's correlation coefficient. After making the logarithmic transformation, logistic regression employing a Wald statistic backward stepwise selection was performed. Differences at *P* < 0.05 were considered statistically significant.

## 3. Results

There were 58 males and 57 females who had undergone elective liver resection with intermittent PTC. Majority of patents (*n* = 98, 85.2%) were classified as ASA 1 or 2 (Table 1). HBV or HCV related chronic viral hepatitis was determined in 27 patients (23.5%). Pathologically confirmed cirrhosis was found in 23 patients in the study group. Indications for resection were malignant disease in 62.6% of the patients (*n* = 72) and benign disease in 37.4% (*n* = 43). Giant hemangiomas and hydatid cyst or alveolar hydatid disease were leading benign causes for liver resection, whereas hepatocellular carcinoma (HCC) and liver metastasis from colorectal carcinoma were leading malign causes for liver resection (Table 1). Double primary liver malignancy (HCC and intrahepatic cholangiocarcinoma) was determined in one patient after pathological examination of the resection material. Major hepatectomy was performed in 26 patients (22.4%) (Table 2). Reresection was performed in one patient for liver metastasis from colorectal carcinoma (two times and interval time between two resection were 14 months). Mean liver ischemia period during liver resection was 27.1 minutes (±13.9 minutes). Cumulative ischemia period of the liver reached up to 60 minutes in 7 of 115 patients. With the use of intermittent PTC, 23.1% of major liver resections (*n* = 6/26) and 81.1% of less extensive liver resections (*n* = 73/90) were performed without any blood transfusion.

The mortality rate was 1.7% (*n* = 2) after hepatectomy. The causes of death were pulmonary embolism after segment 7 resection (*n* = 1) and pneumonia after left hepatectomy (*n* = 1). The morbidity rate was 22.6% (*n* = 26) after hepatectomy (46.2% for major hepatectomy (*n* = 12/26), 18.2% for sectorectomy (*n* = 2/11), 22% for segmentectomy (*n* = 11/50), and 3.4% for nonanatomical subsegmentary liver resection (*n* = 1/29)). The causes and the severity of postoperative complications were summarized in Table 3. Only one patient with Child A cirrhosis was faced with postoperative liver failure after left hepatectomy for hepatocellular carcinoma. Liver failure recovered after 6 cycles of plasmapheresis and medical support.

### 3.1. Biochemical Evaluation of Hepatocyte Injury and Ischemic Insult

**Figure 1:**
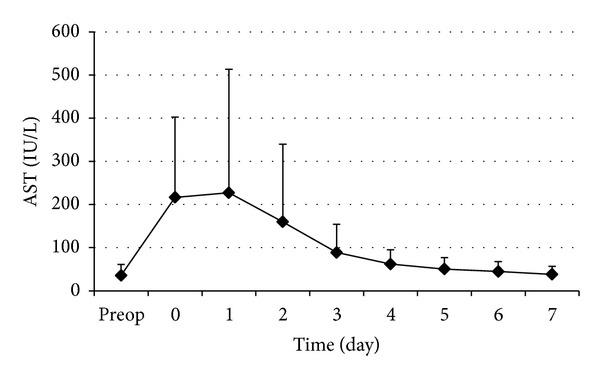
Perioperative changes of serum AST. Data are expressed as mean ± SD.

**Figure 2:**
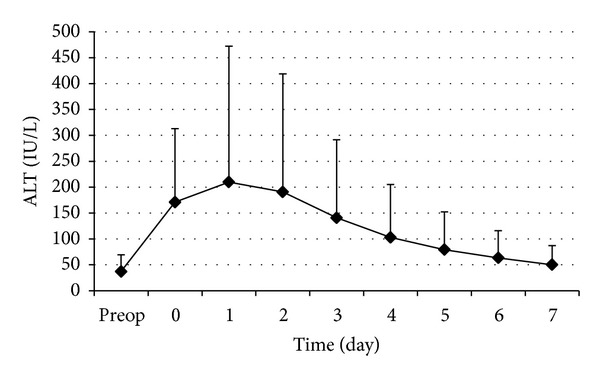
Perioperative changes of serum ALT. Data are expressed as mean ± SD.

**Figure 3:**
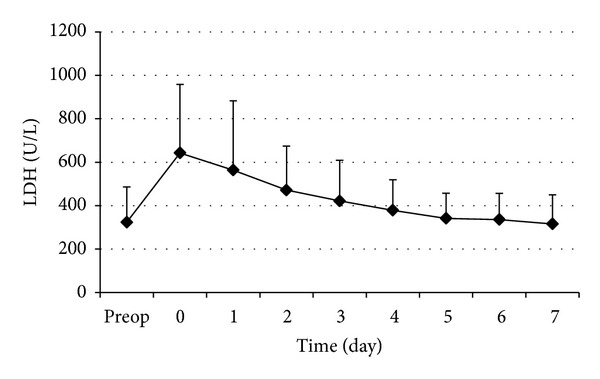
Perioperative changes of serum LDH. Data are expressed as mean ± SD.

The changes in perioperative serum AST and ALT are shown in Figures 1 and 2, respectively. The postoperative serum AST and ALT levels rose rapidly to a peak on day 1 and then decreased gradually in the first postoperative week. The curves of the perioperative serums AST and ALT showed no marked difference. Cumulative clamping time (*t* = 3.61,  *P* < 0.001) and operation time (*t* = 2.38,  *P* = 0.019) were significantly correlated with D-AST (Table 4). Cumulative clamping time (*t* = 5.16,  *P* < 0.001) was significantly correlated with D-ALT (Table 4). The changes in perioperative serum LDH are shown in Figure 3. The postoperative serum LDH levels rose rapidly to a peak on day 0 (day of operation) and then decreased gradually in the first postoperative week. Operation time (*t* = 5.81,  *P* < 0.001) was significantly correlated with D-LDH (Table 4).

### 3.2. Postoperative Alteration on Cholestatic Enzymes

**Figure 4:**
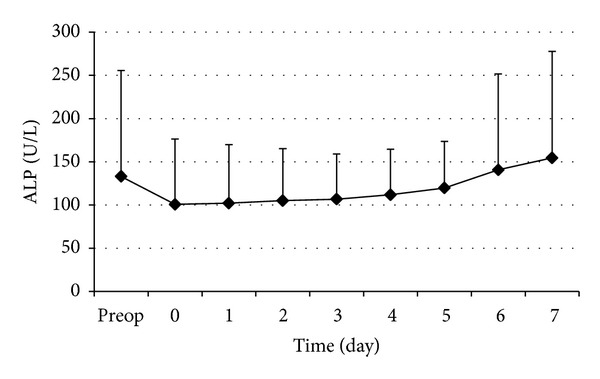
Perioperative changes of serum ALP. Data are expressed as mean ± SD.

**Figure 5:**
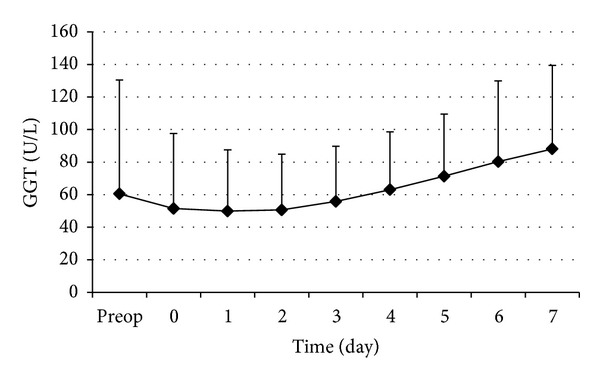
Perioperative changes of serum GGT. Data are expressed as mean ± SD.

The changes in perioperative serums ALP and GGT are shown in Figures 4 and 5, respectively. The postoperative serum ALP and GGT levels decreased gradually after operation and increased gradually after the 2nd postoperative days.

### 3.3. Postoperative Hepatic Functional Reserve

**Figure 6:**
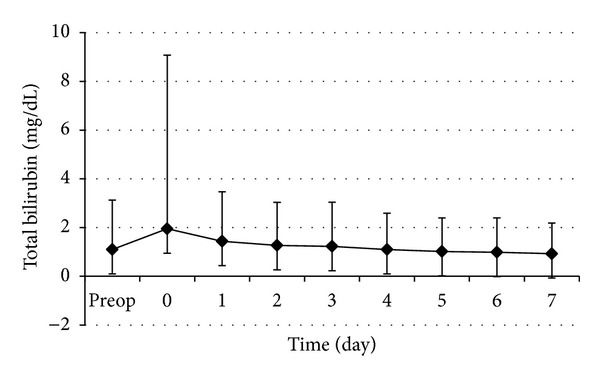
Perioperative changes of serum total bilirubin. Data are expressed as mean ± SD.

**Figure 7:**
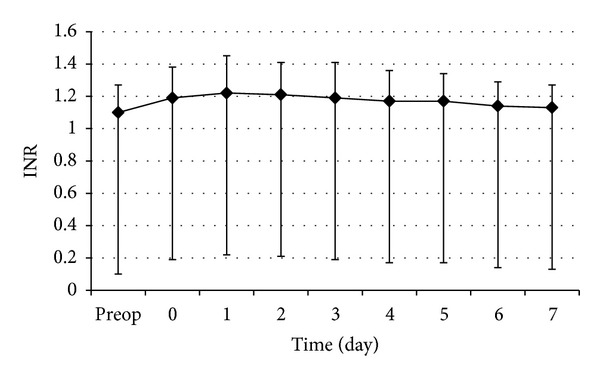
Perioperative changes of serum INR. Data are expressed as mean ± SEM.

The changes in perioperative serum total bilirubin and INR are shown in Figures 6 and 7, respectively. The perioperative serum total bilirubin rose gradually after operation and then decreased in 2 or 3 days. The perioperative INR rose very gradually after operation. The recovery of INR levels was observed as nearly flat curving graphic at the end of first postoperative week.

## 4. Discussion

It is now accepted that liver parenchyma is more tolerant to prolonged continuous normothermic ischemia than to the consequences of massive bleeding and blood transfusions [7, 12–14]. The first priority is therefore to reduce intraoperative blood loss. In this series, 68% of liver resections were performed without any blood transfusion, and this could be achieved with the use of intermittent PTC.

PTC (Pringle maneuver) is the oldest method of hepatic vascular control [15]. The PTC is performed by encircling the hepatoduodenal ligament with a tape and then applying a tourniquet (we also preferred tourniquet) to or a vascular clamp until the pulse in the hepatic artery disappears distally. An aberrant left hepatic artery originating from the left gastric artery should also be occluded if present [7]. After pedicle clamping, a moderate decrease in venous return due to pooling of blood in the mesenteric basin results in a 10% decrease in the cardiac index. Simultaneously, a sympathetic reflex produced by clamping causes a 40% increase in systemic vascular resistance and a 40% increase in mean arterial pressure. Unclamping of the hepatic pedicle leads to a transient decrease in blood pressure because of deactivation of the above-mentioned reflex [16–18]. PTC is generally well tolerated because caval flow is not interrupted and specific anesthetic management is not required [7]. A number of clinical studies have established 60 minutes as the safe duration of continuous PTC under normothermic conditions for both normal and pathologic (mainly cirrhotic) livers [7, 12, 19, 20]. However, it has been reported that continuous PTC has some potential drawbacks. These include portal vein emboli, spontaneous rupture of the spleen [21], induction of hepatic ischemia-reperfusion (IR) injury [22], and splanchnic congestion [13].

The process of warm IR injury involves activation of immune pathways and is dominated by hepatocellular injury. There are 2 distinct phases that occur in warm IR injury. The initial phase is defined as the period less than 2 hours after reperfusion and the late phase of injury, which occurs at 6 to 48 hours after reperfusion [13, 23]. The early phase is marked by activation of immune cells and production of oxidant stress; the later injury is mediated by neutrophil accumulation and hepatocellular injury. In addition to oxidant-mediated damage, the production of cytokines and chemokines also plays a key role in the pathogenesis of IR injury locally and systemically [24]. To minimize adverse effects of continuous PTC, intermittent PTC method has been evolved [7]. It has been shown that the intermittent PTC reduces splanchnic congestion and decreases hepatic IR injury [25]. The initial cycle of clamping/unclamping during intermittent PTC could have a preconditioning hepatoprotective effect [7]. Although the exact mechanisms are not completely understood, the protective effects of ischemic preconditioning include inhibition of apoptosis via the decrease of Kupffer cell activation (activated Kupffer cells release TNF-a, which binds to the TNF-R1 receptor of hepatocytes and initiates the apoptotic process), activation of polymorphonuclear leukocytes, preservation of cellular adenosine triphosphate content, and release of substances such as adenosine and nitric oxide by the ischemic tissue which protect the liver against the subsequent prolonged ischemia [26–28]. Another technical advantage of intermittent PTC is that intermittent release of the portal clamp allows gradual hemostasis over smaller transection areas [29, 30]. However, repeated clamp removal during intermittent PTC may result in fluctuations of systemic blood pressure, multiple episodes of hepatic IR injury, and repeated bleeding from the transection surfaces. However, prospective clinical studies proved the hepatoprotective effect of intermittent PTC [30–32]. Also intermittent PTC permits a significant increase (almost doubling) of the ischemia times that can be achieved with continuous PTC. It can be safely applied up to 120 minutes in both normal and impaired livers [7, 31, 33, 34]. The proven effectiveness of intermittent PTC in reducing bleeding together with its hepatoprotective profile encourages wide application of the method. Application of intermittent PTC under low CVP reduces bleeding during parenchymal transection and during unclamping [33, 35–37]. A low perioperative CVP has been suggested to limit blood loss during liver resection [38, 39]. By lowering the pressure inside the inferior caval vein, the hepatic venous pressure and, thus, the hepatic sinusoidal pressure would drop, possibly resulting in less bleeding during resection [38].

The biochemical investigation of liver IR injury has been a well-evolved issue [40–43]. Alteration on liver transaminase levels reflects hepatocellular injury, whereas accumulation of lactate reflects the severity of tissue ischemia. Postoperative transaminase levels may be related to the volume of liver resection. Clavien and colleagues reported that, for matched patients with the same ischemia time, patients with extended liver resections had lower postoperative peak AST levels than patients with smaller resection volumes. Smaller remnant liver masses might have been associated with lower postreperfusion serum AST and ALT levels than larger residual liver volumes [44]. In a recent study from Japan, it was claimed that the cirrhotic liver releases smaller amounts of aminotransferase than normal liver after IR [45]. The alteration of transaminase levels after liver resection with intermittent PTC in this study seems comparable with the previous studies [46–48]. In the current study, age, gender, BMI, and ASA score of patients, the presence of chronic viral hepatitis or cirrhosis, etiology (benign/malign) for liver resection, the extension of liver resection, intraoperative bleeding amount, and transfusion requirement were found to be unrelated with the postoperative liver injury. Our strict exclusion criteria for patients with chronic liver disease might be a factor for better tolerance of cirrhotic patients to normothermic intermittent inflow occlusion. As well demonstrated in the literature, cumulative clamping time is a major determinant of liver injury due to PTC. In addition to cumulative clamping time, the adverse effects of long operation time on the liver functions were also demonstrated in our study.

Despite the general opinion on the level of alkaline phosphatase and gamma-glutamyltransferase levels not being indicators of liver function and not useful tools to predict liver function after hepatectomy, the postoperative alteration curves of both parameters showed similar characteristics in the first postoperative week. However, postoperative ALP and GGT alteration curves in patients presented with jaundice (Klatskin tumor of gallbladder carcinoma) were discordant from other patients. In jaundiced patients requiring surgery for tumor resection, biliary drainage is suggested before hepatic resection for the elimination of negative effects of cholestasis in liver [49]. The studies from Japan insisted that radical surgery be performed after complete recovery from jaundice; therefore, we performed liver surgery after decrement of total bilirubin level under 2.0 mg/dL. According to this policy, the alteration of bilirubin levels after liver resection might be demonstrated in a common curve without the division of patients with obstructive jaundice or not. When compared to results of Scatton et al., the alteration curve of bilirubin levels in the current study was shown to have similar characteristics.

The absence of randomization and the heterogeneity of the group were main limitations of the study. The effects of comprehensive comorbidities on the development of IR injury may not be shown clearly in such a small study population. Preoperative hospital stay of patients with obstructive jaundice was longer than other patients in the study, at least 3 week. Therefore, to impede any bias on this issue, we analyzed postoperative hospital stay of patients rather than analysis of overall hospital stay. However, all operations were performed by the same surgical team with uniform surgical technique or surgical trauma.

In conclusion, according to our experience, intermittent PTC is an easily applied, flexible method with the inherent risk of bleeding during the reperfusion periods. The morbidity and mortality rates, bleeding amounts, and hospital stay in the current study were found comparable with the previous studies. Intermittent PTC with low CVP permits execution of complex, time consuming resections even in patients with an abnormal liver. Intermittent PTC can be safely applied up to 60 minutes in both normal and impaired livers.

## Figures and Tables

**Table 1:** Patient characteristics.

Variable	Number (%)
Age (mean, ±SD)	55 (±13.7)
Gender	
Male	58 (50.4)
Female	57 (49.6)
BMI (kg/m^2^) (mean, ±SD)	26.5 (±5.2)
Comorbidities	
Presence of anemia	3 (2.6)
Diabetes	16 (13.9)
Systemic arterial hypertension	18 (15.7)
Coronary artery disease	6 (5.2)
Chronic pulmonary disease	2 (1.7)
ASA score	
1-2	98 (85.2)
3-4	17 (14.8)
Chronic HBV infection	23 (20)
Chronic HCV infection	4 (3.5)
Presence of cirrhosis	23 (20)
Benign liver tumors	43 (37.4)
Giant hemangioma	27 (23.5)
Hydatid cyst of the liver	2 (1.7)
Alveolar hydatid disease	6 (5.2)
Hepatolithiasis	4 (3.5)
Granulomatous lesions	4 (3.5)
Malignant liver tumors	72 (62.6)
HCC	32 (27.8)
Metastasis from colorectal carcinoma	22 (20)
Klatskin tumor	8 (7)
Gallbladder cancer	6 (5.2)
ICC	2 (1.7)
Metastasis from breast carcinoma	2 (1.7)
Hemangiosarcoma of the liver	1 (0.9)

BMI: body mass index, ASA: American Society of Anesthesia; HBV: hepatitis B virus; HCV: hepatitis C virus; HCC; hepatocellular carcinoma; ICC: intrahepatic cholangiocarcinoma; SD: standard deviation.

**Table 2:** Intraoperative and postoperative parameters of the study group.

Variable	Number (%)
Major hepatectomy	26 (22.4)
Right hepatectomy	5 (4.3)
Extended right hepatectomy	5 (4.3)
Left hepatectomy	11 (9.5)
Extended left hepatectomy	5 (4.3)
Sectorectomy	11 (9.5)
Posterior sectorectomy	1 (0.9)
Medial sectorectomy	1 (0.9)
Lateral sectorectomy	9 (7.8)
Segmentectomy	50 (43.1)
Nonanatomical subsegmentary liver resection	29 (25)
Reresection	1 (0.9)
Portal triad clamping period (minutes) (mean, ±SD)	27.1 (±13.9)
Additional surgery	2 (1.7)
Operative time (minutes) (mean, ±SD)	207.7 (±93.3)
Intraoperative bleeding amount (milliliters) (median, min–max)	382 (20–2000)
Intraoperative or postoperative blood transfusion	37 (32)
Intraoperative or postoperative fresh frozen plasma transfusion	93 (80.2)
Morbidity	26 (22.6)
Reoperation	2 (1.7)
Mortality	2 (1.7)
Requirement of ICU care	4 (3.5)
Length of postoperative hospital stay (days) (mean, ±SD)	11.5 (±7.1)

ICU: intensive care unit; SD: standard deviation.

**Table 3:** Distribution of postoperative complications according to Dindo's gravity index.

Type of complication	Severity of complication	*N* (%)
Atelectasis	Dindo I	4 (3.5)
	Dindo IIIa	1 (0.9)
Pleural effusion	Dindo I	2 (1.7)
	Dindo II	4 (3.5)
	Dindo IIIa	3 (2.6)
Pneumonia	Dindo II	4 (3.5)
	Dindo IVb	1 (0.9)
Pulmonary embolism	Dindo V	1 (0.9)
Wound infection	Dindo I	7 (6)
	Dindo IIIb	1 (0.9)
Biliary leak	Dindo II	4 (3.5)
	Dindo IIIb 2	2 (1.7)
	Dindo IIIa 2	2 (1.7)
Liver failure	Dindo IVa	1 (0.9)

**Table 4:** Factors affecting delta-aspartate aminotransferase, delta-alanine aminotransferase, and delta-lactate dehydrogenase.

	Parameter estimate	Standard error	95% confidence interval	*t*	*P*
Factors affecting D-AST					
Cumulative clamping time	0.010	0.003	0.005–0.015	3.609	<0.001
Operative time	0.0010	0.0004	0.0002–0.002	2.386	0.019
Factors affecting D-ALT					
Cumulative clamping time	0.016	0.003	0.010–0.022	5.165	<0.001
Factors affecting D-LDH					
Operative time	0.001	0.0002	0.0007–0.0016	5.813	<0.001

D-AST: delta-aspartate aminotransferase; D-ALT: delta-alanine aminotransferase; D-LDH: delta-lactate dehydrogenase.
